# Acute osteomyelitis of the humerus mimicking malignancy: *Streptococcus pneumoniae* as exceptional pathogen in an immunocompetent adult

**DOI:** 10.1186/1471-2334-13-266

**Published:** 2013-06-05

**Authors:** Peter M Prodinger, Hakan Pilge, Ingo J Banke, Dominik Bürklein, Reiner Gradinger, Thomas Miethke, Boris M Holzapfel

**Affiliations:** 1 Clinic for Orthopaedics and Sports Orthopaedics, Klinikum Rechts der Isar, Technical University Munich, Ismaninger Straße 22, D-81675, Munich, Germany; 2 Department of Orthopaedics, University Clinic Duesseldorf, Moorenstr. 5, D-40225, Duesseldorf, Germany; 3 Department of Orthopaedic Surgery, Koenig-Ludwig Haus, Julius-Maximilians-University of Wuerzburg, Brettreichstr. 11, D-97074, Wuerzburg, Germany; 4 Institut für medizinische Mikrobiologie, Immunologie und Hygiene, Klinikum Rechts der Isar, Technische Universität München, Ismaninger Straße 22, D-81675, Munich, Germany

**Keywords:** Acute osteomyelitis, Haematogenous, *S. pneumoniae*, Long bones, Immunodeficiency, Osteomyelitis of the humerus

## Abstract

**Background:**

Chronic osteomyelitis due to direct bone trauma or vascular insufficiency is a frequent problem in orthopaedic surgery. In contrast, acute haematogenous osteomyelitis represents a rare entity that almost exclusively affects prepubescent children or immunodeficient adults.

**Case Presentation:**

In this article, we report the case of acute pneumococcal osteomyelitis of the humerus in an immunocompetent and otherwise healthy 44-year-old male patient presenting with minor inflammation signs and misleading clinical features.

**Conclusions:**

The diagnosis had to be confirmed by open biopsy which allowed the initiation of a targeted therapy. A case of pneumococcal osteomyelitis of a long bone, lacking predisposing factors or trauma, is unique in adults and has not been reported previously.

## Background

*S. pneumoniae*, or pneumococcus, is a Gram-positive, alpha-haemolytic and bile soluble diplococcus. This bacteria is facultative anaerobe and a member of the genus *Streptococcus*. *S. pneumonia*, it’s the most common cause of bacterial meningitis in adults and children, and is one of the top two isolates found in otitis media [1]. Bacteremia rarely leads to pneumococcal osteomyelitis or septic arthritis in premature infants and neonates [2] or in immunodeficient adults [3]. *S. pneumoniae* has never been associated to acute osteomyelitis in immunocompetent patients so far.

In the following we present the case of acute osteomyelitis caused by pneumococci in a healthy adult patient. Due to the nonspecific nature of the clinical and radiological features presented, diagnosis had to be secured by open biopsy. This enabled us to initiate targeted antibiotic treatment. In this article we furthermore review the recent literature and discuss actual therapeutic strategies for acute, haematogenous osteomyelitis.

## Case presentation

A 44-year-old otherwise healthy male was referred to the orthopaedic unit with a four week history of minor pain in the right upper arm. The onset of pain was accompanied by an episode of flu-like symptoms that subsided immediately. Later, an indolent swelling was noted on the lateral side of the upper arm. Increasing numbness in the lateral forearm and hand finally encouraged the patient to consult his practitioner.

**Figure 1:**
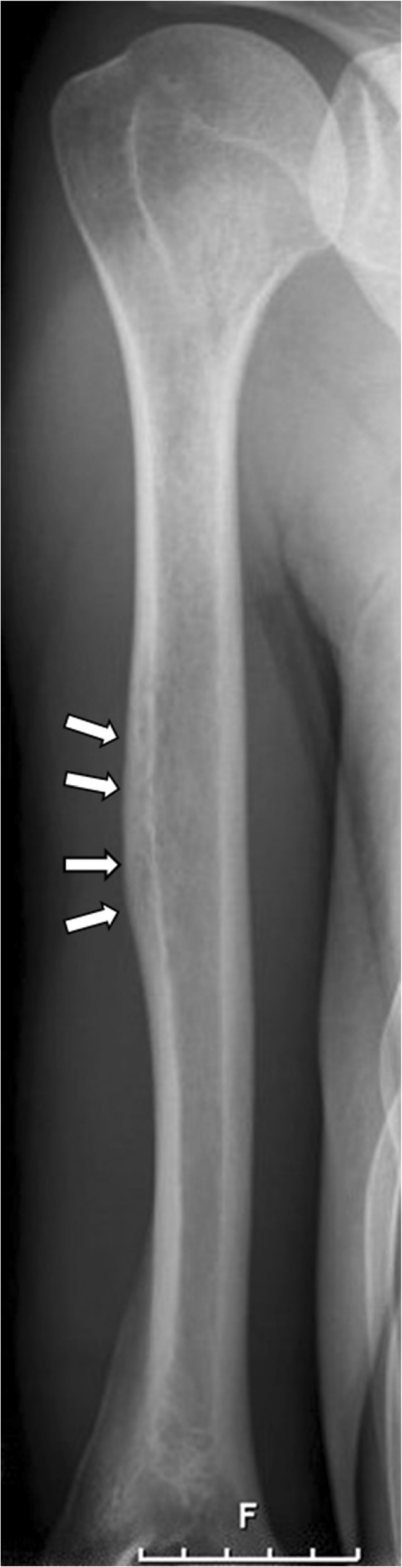
Radiograph of the right humerus: cortical disaggregation suggesting a permeative process which can be seen at the lateral, diaphyseal corticalis (arrows).

**Figure 2:**
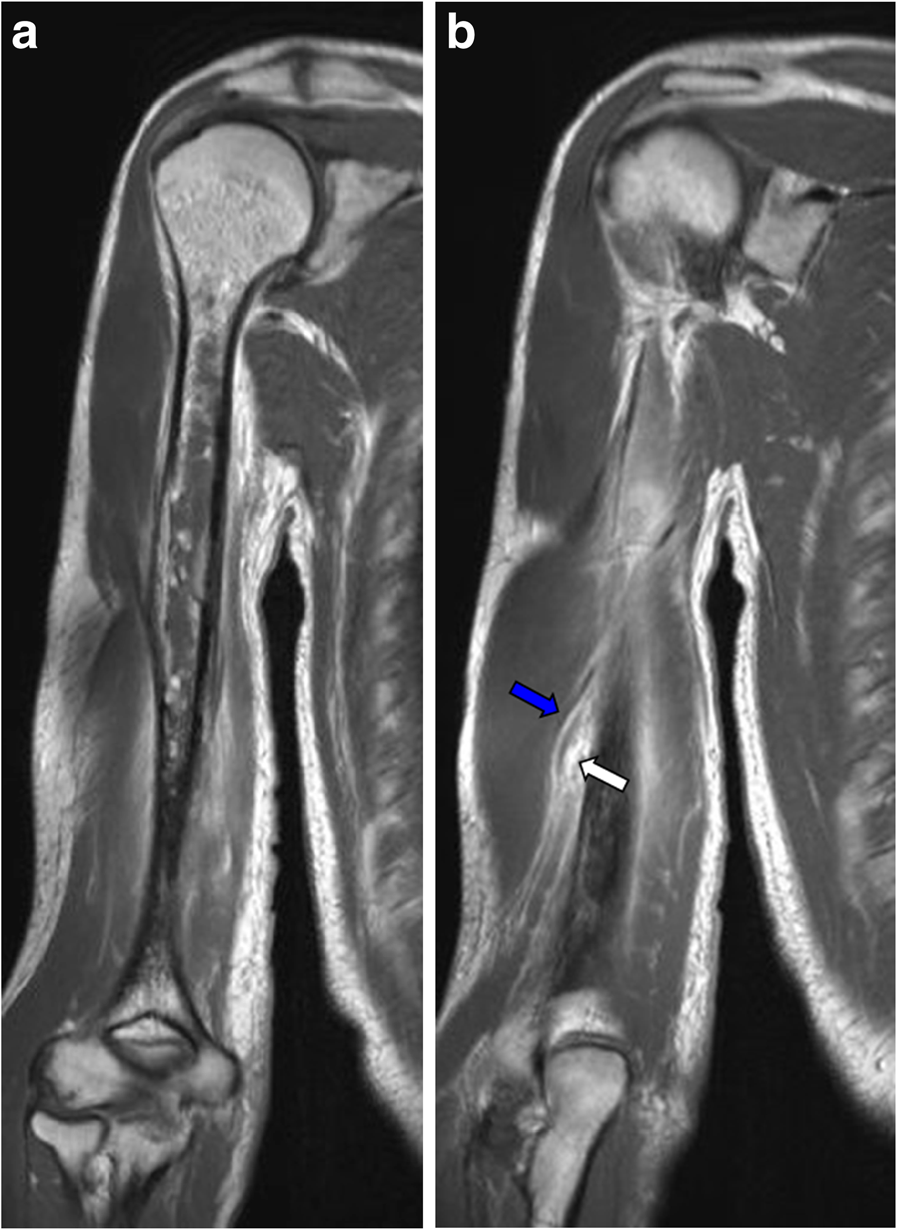
MRT of the right humerus: T1 with Gadolinium (*tse2d1rs3), coronar views. a: Cortical infiltration of the middle third of the diaphysis. The marrow is replaced by extensive edema, only few hyperintense nodules are left. **b**: Extraosseal soft tissue reaction (white arrow) affecting the radial nerve (blue arrow).The differential diagnoses are: acute osteomyelitis, Ewing sarcoma or osteosarcoma.

The physical examination indicated a palpable, indolent soft tissue tumour located at the dorso-lateral, distal side of the right humerus. The localisation of the numb area reported by the patient corresponded to area of the radial nerve´s sensible innervation. A blood sample was taken and revealed mild anaemia (Hb 13,0 mg/dl [norm: 14–18 mg/dl]), moderate leucocytosis (11,46 G/l [norm: 4,0–9,0 G/l]) and slightly elevated CRP (1,8 mg/dl [norm: <0,5 mg/dl]) and CK (232 U/l [norm: <174 U/l]). Subsequently, X-rays of the right humerus showed cortical disaggregation and extraosseal soft tissue swelling (Figure 1) and were highly indicative for malignancy. MRI of the right upper arm revealed an inhomogenous tumour affecting the dia- and metaphysis of the right humerus (normal marrow displaced, T1 and T2 hyperintense noduli). The corticalis was infiltrated and permeated, leading to a small extraosseal tumour mass (15 × 6 mm) on the dorsolateral side of the bone. This tumour mass led to a compression of the radial nerve (Figure 2).

**Figure 3:**
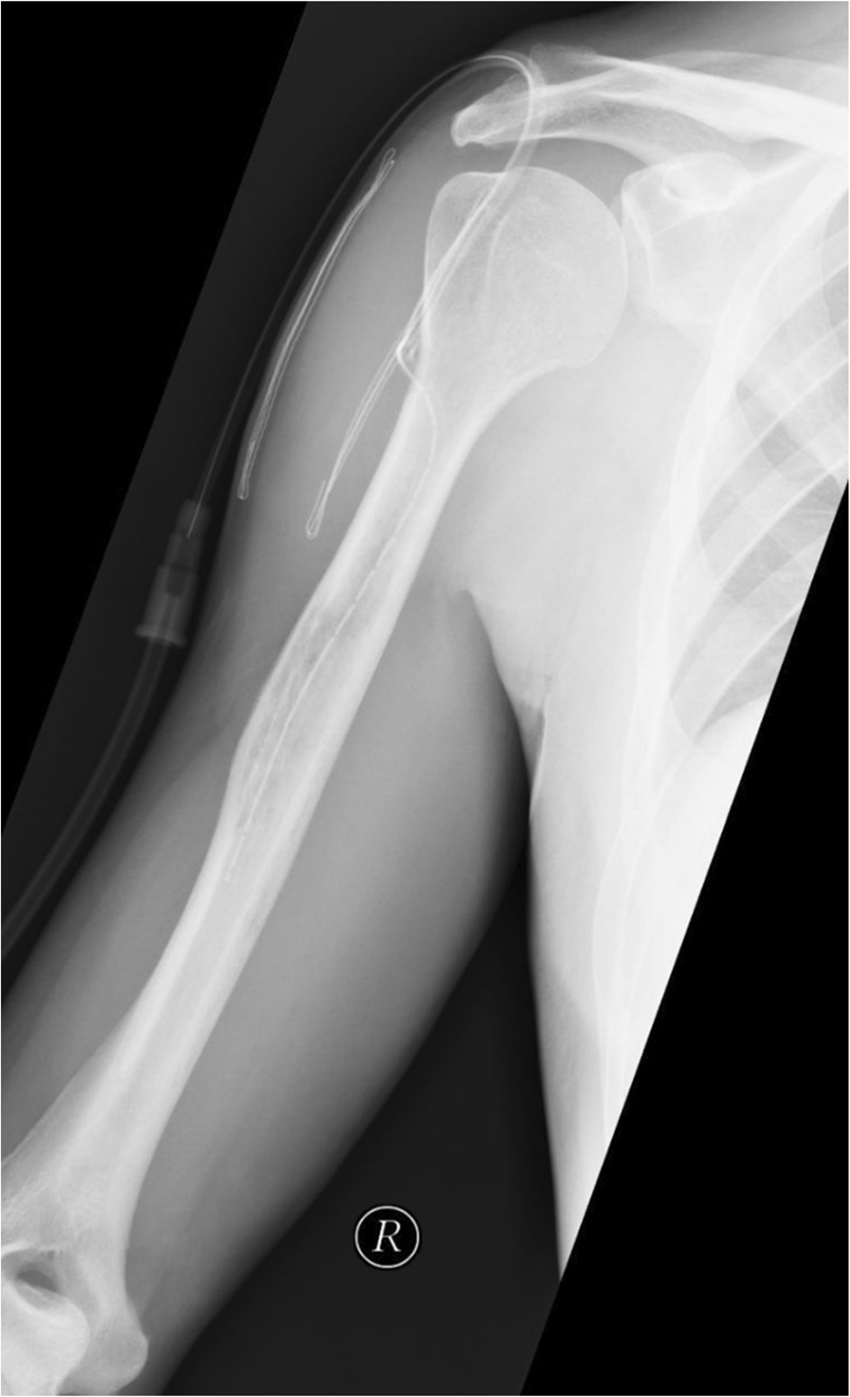
Radiograph of the right humerus, anterioposterior direction, postoperative status: drainage inserted via osseal window.

Therefore, an open biopsy of the proximal diaphysis of the right humerus was performed. After drilling into the bone, spontaneous extrusion of purulence was evident. Microbiological samples were takenand the marrow space rinsed and drained (Figure 3). Calculated antibiotic treatment with Cefuroxim (4,5 g/d i.v.) was initiated and switched to Ceftriaxone (2 g/d i.v.) after *Streptococcus pneumoniae* was identified. Screening for infections causing immunodeficiency (HIV, Hep. B, Hep. C, EBV, CMV, Mycobacteria) was negative. Congenital asplenia was ruled out by a routine ultrasound check-up. A pharyngeal swab did not show local colonisation with *S. pneumoniae* and pathologies of the lungs including clinical unapparent pneumonia were excluded by chest X-ray.

The patient was discharged on the 10^th^ postoperative day showing no signs of infection. Antimicrobiotic treatment was changed to Cefuroxim 1000 mg/d p.o. and continued for four weeks. Following check-ups after 30 days, 3 months and 9 months did not result in any pathological finding. Clinically, the scar tissue showed no signs of persistent infection, the patient did not report any pain in the shoulder or arm and the function of the limb was normal. The white blood cell count was normal at discharge and stayed normal during the whole follow up. CRP at discharge was slightly elevated (1,1 mg/dl [norm: <0,5 mg/dl]), at 30 days it decreased to 0,3 mg/dl and stayed below 0,5 mg/dl thereafter. We performed X-rays of the humerus after 30 days and a control MRI-scan after 3 months. Both revealed no signs of osteonecrosis complicating the infection.

Acute osteomyelitis in adults is rare and generally linked to immunodeficiency, diabetes, vasculopathy, precedent trauma or surgery [4]. A haematogenous spread of bacteria leading to osteomyelitis is mostly seen in prepubescent children or elderly patients and is characterised by nidation of bacteria within bones without direct trauma or open wound [4]. Haematogenous osteomyelitis is well known as being the most common musculoskeletal complication in patients with HIV or AIDS [5]. However, in the literature there is not one single case of acute, haematogenous pneumococcal osteomyelitis affecting long bones in an otherwise healthy adult patient who lacks trauma history or immunodeficiency.

Bacteria causing haematogenous osteomyelitis reflect their frequency in the blood as a function of age [6,7]. Thus, organisms most commonly encountered in infants and neonates include *Staphylococcus aureus*, group B streptococci, coagulase-negative staphylococci and different other streptococci [7-10]. With age, *S. aureus* dominates. In elderly people, commonly subject to gram-negative bacteremia, osteomyelitis - especially vertebral osteomyelitis - can also be caused by this group of microorganisms [11]. Pneumococci as uncommon infectious agent of the bone are rarely known to cause vertebral osteomyelitis [4,12-14]. Pneumococcal bone manifestations are generally linked to clinically apparent upper and lower respiratory tract infections caused by *S. pneumoniae*, sickle cell anemia [4] or direct bone trauma [12,13]. Asplenia is also associated with an increased risk of severe pneumococcal infections, thus predisposing for affecting the bone via haematogenous spread [15]. All mentioned co-factors were missing in this particular case.

The clinical features of haematogenous, acute osteomyelitis in long bones are typical: chills, fever, and malaise reflect the bacteremic spread of microorganisms as shown by positive blood cultures. Strong pain and local swelling are hallmarks of the local infectious process. The clinical examination should include a search for bone tenderness on palpitation [4]. By contrast, in our case the clinical symptoms were a painless swelling and nerve disfunction, being strongly suggestive for a continuously growing process commonly found in primary bone tumours or metastases.

Distinguishing between subclinical haematogenous osteomyelitis and primary or metastatic bone tumours by imaging can be challenging [16,17]. The earliest radiographic changes in osteomyelitis are swelling of soft tissue, periosteal thickening or focal osteopenia, which is frequently observed in malignant bone tumours as well [17,18]. The typical appearance of acute osteomyelitis in MRI is a localized area of abnormal marrow with decreased signal intensity in T1-weighted images and increased signal intensity in T2-weighted images [16], as shown in our case. MRI is highly sensitive in the detection of pathologic changes in bone marrow and can give precise information about the localization and extent of an infection, but it has not yet provided pathognomonic findings for osteomyelitis [17]. Thus, the presence of a primary or metastatic bone tumour had to be excluded by open biopsy.

Recommendations concerning antibiotic treatment of hematogenous osteomyelitis differ regionally according to the local type and resistance-pattern of the causing bacteria. In Germany, the Paul-Ehrlich society would favour a combination of 2^nd^ generation cephalosporin with clindamycin as first-line calculated therapy in adults, assuming that the infection is most likely be caused by *S. aureus* or β-haemolysing streptococci [19]. In pediatric osteomyelitis, up to 5% of all cases were identified to be caused by S*. pneumoniae*[20]. *S. aureus* as the top-isolate and also pneumococci do respond to a first line antibiotic treatment with a 1^st^ generation cephalosporin, clindamycin or high-dose penicillin G according to most authors [20-22]. Notably, *S. pneumoniae* has been shown to have a lower minimal inhibitory concentration (MIC) to penicillin than other streptococci (e.g. *S. pyogenes*), though the clinical impact is questionable and depends on the breakpoint used. Altogether, resistant strains are not frequent and account for only 0.24% [23]. Unlike in adult osteomyelitis, no extensive surgical therapy is required in children. However, it is also important to gain representative tissue samples from the bone to be able to initiate targeted therapy after culture [18]. The duration of the antibiotic treatment should be 1–3 months. Recent studies challenged this approach and suggest that 20 days of treatment including an initial i.v. period of 2–4 days should be sufficient to control the infection [22].

Invasive pneumococcal infections in adults are rare and generally affect immunodeficient patients. Resistance-patterns to antibiotics vary regionally and depend on the breakpoint of the MIC-definition used. In most countries high-dose parenteral penicillin G or cefuroxim are recommended as first-line therapy. Cefriaxone has been shown to have one of the lowest MICs making this substance effective, especially in the treatment of severe pneumococcal infections [24,25]. Specific treatment recommendations for pneumococcal osteomyelitis in adults are completely missing. On the basis that ceftriaxone is a very effective substance against other severe pneumococcal infections in pediatric and aduld patients [2,25] we adopted this substance once the bacteria was identified and were able to control the symptoms within a short period. Retrospectively, one can assume that high-dose penicillin G would have had the same efficacy. In addition, the continuation of cefuroxime, probably combined with clindamycin would have been another reasonable alternative.

According to recent treatment guidelines, the initial i.v. therapy of hematogenous osteomyelitis should last for 1–4 weeks and should be continued by p.o. therapy for 2–6 weeks [19]. Since our patient received i.v. therapy for only 10 days, the total duration of therapy was rather short. However, since all inflammatory parameters were within the normal range and clinical inspection did also not reveal symptoms of infection 30 days after discharge, we stopped therapy at this time point. As given above, therapy for uncomplicated cases of osteomyelitis in children is even shorter [26,27]. 2^nd^ and 3^rd^ generation cephalosporins were identified as major risk factors for developing pseudomembranotic colitis, especially in combination with clindamycin [28,29]. We therefore decided to use the 2nd generation cephalosporin alone for ambulatory treatment.

## Conclusions

Haematogenous spreading of bacteria can cause acute osteomyelitis in prepubescent children and immunodeficient adults. Frequently, the vertebral bodies are affected. Manifestations in long bones seem to be exceptional in immunocompetent patients but should be considered. *S. pneumoniae* is an unexpected bacterial agent in mature patients showing absence of respiratory tract infections or pharyngeal colonisation. Non-specific clinical and imaging findings necessitate open biopsy. Microbiological and pathologic evaluation of the material obtained should be performed to isolate the causative pathogen and achieve appropriate treatment.

To summarize, not one of the mentioned etiological aspects for developing acute, haematogenous osteomyelitis matches the case described and the clinical symptoms were misleading. Nevertheless, close workup of clinical and radiological findings led to the suspicion of acute osteomyelitis, which was confirmed and completed by the microbiological results. Therefore, this case approves the surgical principle: “Biopsy what you culture and culture what you biopsy!”

## Consent

Written informed consent was obtained from the patient for publication of this Case report and any accompanying images. A copy of the written consent is available for review by the Editor-in-Chief of this journal.

## Competing interests

The authors declare that they have no competing interests.

## Authors’ contributions

PMP and BMH drafted and wrote the manuscript and provided surgical guidance of the patient. HP, IJB and DB were involved in the clinical guidance of the patient and contributed to figures and discussion. TM performed the microbiological examinations and participated in the conception of the manuscript. RG provided surgical guidance and coordinated the work. All authors read and approved the final manuscript.

## Pre-publication history

The pre-publication history for this paper can be accessed here:

http://www.biomedcentral.com/1471-2334/13/266/prepub
